# Feline eosinophilic sclerosing fibroplasia associated with T-/natural killer-cell lymphoma

**DOI:** 10.1177/03009858241281911

**Published:** 2024-09-25

**Authors:** Andrea Klang, Christof A. Bertram, Taryn A. Donovan, Linden E. Craig, Ingrid Walter, Birgitt Wolfesberger, Brigitte Degasperi, Elisabeth Baszler, Barbara C. Rütgen, Sabine E. Hammer, Andrea Fuchs-Baumgartinger

**Affiliations:** 1 University of Veterinary Medicine Vienna, Vienna, Austria; 2 The Schwarzman Animal Medical Center, New York, NY; 3 The University of Tennessee, Knoxville, TN

**Keywords:** cat, clonality testing, gastrointestinal tract, immunohistochemistry, lymphoma, lymphoproliferative disorders, natural killer cells

## Abstract

Feline eosinophilic sclerosing fibroplasia (FESF) is a proliferative, inflammatory disease of the gastrointestinal tract and other sites, uncommonly diagnosed in the cat. This entity of uncertain etiology typically presents as a progressive mass lesion, mimicking a neoplastic process. In this case series, we present 17 cases of FESF associated with intralesional lymphoma. Histologic and immunohistochemical characterization of this unique lymphoma revealed that the neoplastic lymphocytes were immunopositive for CD56 and/ or CD3, suggesting a natural killer cell, natural killer T-cell, or T-cell origin. This case series represents the first description of this lymphoma subtype, for which the term eosinophilic sclerosing lymphoma is proposed.

Feline eosinophilic sclerosing fibroplasia (FESF) was first described in 2009 and has been further reported worldwide.^3,4,8,17,24,37^ Typical macroscopic findings in the gastrointestinal tract include a commonly ulcerated intramural mass in the pyloric sphincter, the small intestine, the ileocecocolic junction, or the colon. Mesenteric lymphadenomegaly is a frequent concurrent finding.^3,5,15,24,35^ These lesions have also been described arising outside the abdominal cavity, such as the retroperitoneum^31^ and the cranial mediastinal lymph nodes.^35^ Characteristic histopathologic findings include dense branching collagen trabeculae, interspersed by large reactive fibroblasts, and mixed inflammatory cell infiltrates, predominately eosinophils. Intralesional microabscessation and bacteria are also common findings.^4^ Owing to its proliferative nature, FESF may mimic neoplasia but is believed to be a reactive process to various stimuli or pathogens. To date, there is only 1 case report of FESF with round cell aggregates consistent with a lymphoma.^35^ Here, we present 17 cases of FESF associated with intralesional neoplastic lymphoid cell proliferations, which were characterized via immunohistochemical examination.

## Materials and Methods

Archived surgical biopsy and necropsy samples of 17 cats with a histologic diagnosis of FESF and intralesional lymphoproliferative disease were collected at the Veterinary University of Vienna, Austria (9 cases), the Schwarzman Animal Medical Center, New York, USA (2 cases), and the University of Tennessee College of Veterinary Medicine, Knoxville, USA (6 cases). Sections of formalin-fixed paraffin-embedded tissues were stained with hematoxylin and eosin. Immunohistochemistry was performed on formalin-fixed paraffin-embedded samples for CD3, CD20, CD56, CD57, granzyme B, and MUM1 antigen on an automated immunostainer (Lab Vision AS 360, Lab Vision, Thermo-Fisher Scientific, Fremont, California) (Supplemental Table S1). Horseradish peroxidase polymer (ImmunoLogic, Duiven, The Netherlands) was applied and visualized with DAB Quanto Substrate System (Lab Vision, Thermo-Fisher Scientific). Normal tissues from feline spleen, lymph node, and small intestine were used as a positive control. Antibody specificity for CD56, CD57, and granzyme B in feline lymphoid cells has already been evaluated.^6,10,23^ The primary antibody was omitted from negative controls, and in addition, a feline ileocecal B-cell lymphoma served as a control.

For the polymerase chain reaction (PCR)-based lymphocyte clonality assay, total genomic DNA was extracted from archived surgical biopsy and necropsy tissue samples with 200 µl elution buffer using a commercial kit following the manufacturers’ instructions (E.Z.N.A. Tissue DNA Kit, Omega Biotech, Norcross, Georgia). Genomic DNA concentration and quality were determined using the NanoDrop 2000c spectrophotometer (Thermo-Fisher Scientific, Waltham, Massachusetts) in pedestal mode. The threshold was set to 30 ng/µl with desired 260/280 ratios of 1.8 to 2.0 and 260/230 ratios above or equal 2 (2.0-2.2).^12^ The genomic DNA samples were assayed by amplifying a 189 base pair fragment of the feline *androgen receptor* gene; the *immunoglobulin heavy chain (IGH)-VDJ* gene rearrangements with the primer sets IGH-VDJ, IGH-DJ, Kde, and IGL; and the *T-cell receptor gamma* (*TRG*) *chain VJ* gene rearrangements with the primer sets TRG-J1, TRG-J2, and TRG-J3.^12,22,21,26^ Each PCR reaction was carried out in triplicate including positive and negative PCR controls in each run.^10,12^

After PCR, 10 µl of DNA Dilution Buffer (Qiagen, Hilden, Germany) was added to each PCR reaction and size separated using the QIAxcel Advanced System capillary electrophoresis analyzer with the QIAxcel DNA High Resolution Kit and the QX Alignment Marker 15 bp/1000 bp (Qiagen). The presence and size of obtained PCR products were accurately determined using QIAxcel ScreenGel Software (Qiagen). Identical PCR triplicates verified the reproducibility of the clonality patterns, which were interpreted as described previously.^10,11,16^

## Results

**Figure 1.**
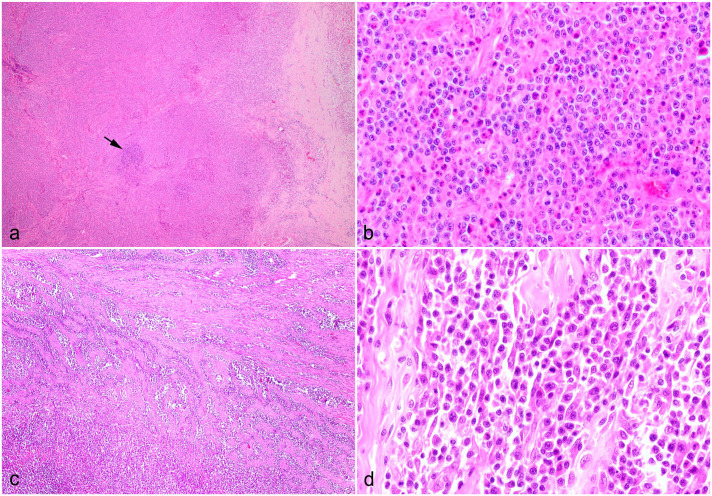
Histological characteristics of feline eosinophilic sclerosing fibroplasia associated lymphoma, cat. Hematoxylin and eosin. (a) Mesenteric lymph node, case 2. The architecture is lost due to neoplastic lymphoid cells and fibroplasia. One residual follicle is still visible in the center (arrow). (b) Higher magnification of (a) demonstrating neoplastic lymphoid cells with medium-sized, oval, and sometimes indented nuclei with a dispersed chromatin, which commonly contain a central prominent nucleolus. Lymphocytes are interspersed by numerous eosinophils. (c) Small intestine, case 3. The tunica muscularis is expanded by trabecular fibrous strands and neoplastic lymphoid cells infiltrating into the lamina propria. (d) Small intestine, case 17. Proliferation of medium-sized neoplastic lymphoid cells with sometimes a centrally located nucleolus, admixed with few eosinophils and fibrous tissue.

**Figure 2.**
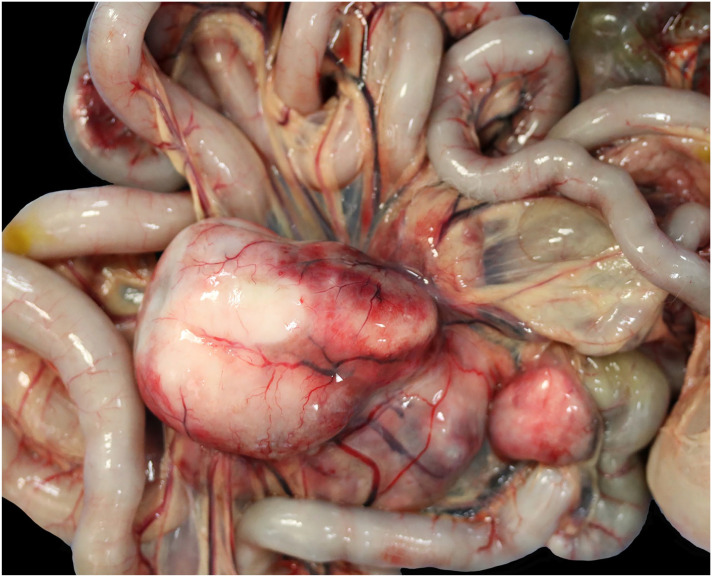
Macroscopic necropsy picture of enlarged mesenteric lymph node from feline sclerosing fibroplasia associated lymphoma, cat, case 10.

The study population comprised 12 of 17 domestic shorthair cats, 3 of 17 domestic longhair cats, 1 of 17 mixed breed cat, and 1 of 17 Maine coon cat; 8 of 17 male castrated and 8 of 17 spayed female animals as well as 1 of 17 intact female cat. The median age of the cats was 10.6 years, ranging from 5 to 15 years. Of 13 cases for which information was available, 9 animals were euthanized and 4 animals died naturally. Nine cats died within 3 months after clinical presentation. The survival time of 4 cats treated with chemotherapy was 4, 9, 10, and 12 months. A review of complete blood count findings revealed that 12 of 15 cats in this case series had peripheral eosinophilia. The lesions were focal or multifocally localized in mesenteric lymph nodes (13 of 17 cases), small intestine (9 of 17 cases), stomach (2 of 17 cases), and liver (1 of 17 cases). Histologically, all gastrointestinal and mass lesions contained a variable degree of fibrosis partially composed of branching collagen trabecular structures and activated fibroblasts (Fig. 1c, d). This finding was accompanied by eosinophilic infiltration of variable severity (Fig. 1b, d). The inflammation ranged from mucosal to transmural and was multifocal, coalescing, or diffuse. Enlarged mesenteric lymph nodes (Fig. 2) contained focal to multifocal eosinophilic inflammation and prominent sclerosis. In addition, lesions were accompanied by variable amounts of lymphoid cells with nuclei ranging from 1.5 to 2 times the diameter of an erythrocyte (interpreted as intermediate sized) with minimal to moderate cellular atypia. These cells contained scant cytoplasm; round to oval and occasionally indented or clefted nuclei with dispersed chromatin; and a single, commonly centrally located, sometimes prominent nucleolus (Fig. 1b, d). The mitotic count ranged from 1 to 19 mitotic figures per 10 high-power field (HPF) with an ocular field number of 22 (0.237 mm^2^) each (1-19 mitotic figures/2.37 mm2). Representative cases with these histopathologic characteristics are depicted in Fig. 1.

In addition, in some animals, lymphoma without FESF lesions was localized apart from the gastrointestinal tract, specifically in the liver, kidney, lung, and cranial mediastinal lymph node. Information about patient specifications, survival times, chemotherapy, and lesion sites are provided in Table 1.

**Table 1.** Signalment, survival time, chemotherapy, and lesion sites in feline eosinophilic sclerosing fibroplasia associated lymphoma.

Case	Breed	Sex	Age (years)	Survival (months)	Chemotherapy	GIT lesion site(s)
1	DSH	mc	10	0.5	no	LN
2	DSH	f	11	12	yes	LN
3	DSH	mc	11	3	no	LN, JE, IL
4	DSH	mc	11	2	no	LN
5	DSH	mc	8	9	yes	LN, SI
6	DSH	fs	11	1	no	LN
7	DSH	mc	7	1	no	LN, SI
8	DLH	fs	12	na	na	LN
9	DSH	fs	10	2	no	LN
10	MaC	fs	12	2	no	LN, IL
11	DSH	fs	14	na	na	LN
12	DLH	mc	8	2	no	JE
13	DLH	mc	12	na	na	ST, DU
14	MB	mc	5	2	no	ST
15	DSH	fs	11	na	na	DU
16	DSH	fs	15	4	yes	LN, JE, LI
17	DSH	fs	12	10	yes	LN, JE

Abbreviations: DLH, domestic longhair; DSH, domestic shorthair; MaC, Maine coon; MB, mixed breed; f, female; fs, female spayed; mc, male castrated; na, information not available; GIT, gastrointestinal; DU, duodenum; IL, ileum; JE, jejunum; LI, liver; LN, mesenteric lymph node; SI, small intestine; ST, stomach.

**Figure 3.**
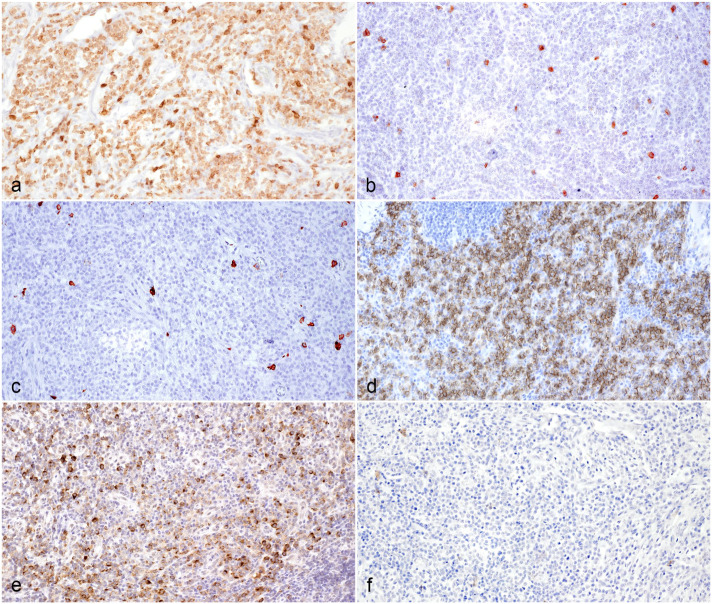
Immunohistochemical characteristics of the predominant phenotype of feline sclerosing fibroplasia associated intestinal lymphoma, cat. Immunohistochemistry (IHC). (a) Neoplastic lymphoid cells demonstrating strong cytoplasmic immunolabeling for CD3, case 1. (b) Negative CD3 immunolabeling of neoplastic lymphoid cells, case 2. (c) Neoplastic lymphoid cells are negative for CD20, case 1. (d) Strong membranous immunolabeling of neoplastic lymphoid cells for CD56, case 2. (e) Moderate to strong cytoplasmic granzyme B labeling of neoplastic lymphoid cells, case 1. (f) Neoplastic lymphoid cells are negative for CD57, case 1.

CD56 immunohistochemistry revealed variable amounts of strong membranous immunolabeling in 14 of 17 cases. Of these 14 CD56^+^ cases, 7 of 14 were CD3^+^ indicating natural killer T-cells (NKT cells) and 7 were CD3^−^ fitting for natural killer (NK) cells. The 3 cases that were immunohistochemically negative for CD56 antigen, demonstrated strong cytoplasmic CD3 immunolabeling. Of these 3 T-cell cases, 2 were granzyme B^−^ and 1 case was granzyme B^+^. Five of the 7 NKT cases as well as 5 of the 7 NK cases were granzyme B^+^. Neoplastic round cell populations were consistently immunohistochemically negative for CD20, CD57, and MUM1 antigen. Characteristics of the most common immunohistochemical phenotypes are depicted in Fig. 3. Detailed results of immunolabeling are shown in Table 2.

**Table 2.** Immunohistochemistry results, mitotic count of proliferating lymphoid cells, lymphocyte clonality PCR testing result for *TRG*, and proposed entity regarding the full analysis pattern in feline eosinophilic sclerosing fibroplasia associated lymphoma.

Cases	Immunohistochemical results						MC	Clonality *TRG*	Proposed entity
	CD3	CD20	CD56	CD57	Granzyme B	MUM1			
1	90% +	−	90% +	−	80% +	−	2	pc	NKT
2	−	−	90% +	−	50% +	−	19	pc	NK
3	90% +	−	70% +	−	50% +	−	4	mc+pc bg	NKT
4	−	−	90% +	−	−	−	13	pc	NK
5	40% +	−	90% +	−	50% +	−	2	pc	NKT
6	40% +	−	80% +	−	−	−	14	oc	NKT
7	80% +	−	90% +	−	−	−	6	pc	NKT
8	70% +	−	50% +	−	80% +	−	18	pc	NKT
9	80% +	−	30% +	−	30% +	−	6	pc	NKT
10	−	−	90% +	−	−	−	2	pc	NK
11	−	−	80% +	−	30% +	−	5	pc	NK
12	70% +	−	−	−	80% +	−	9	bc	T
13	40% +	−	−	−	−	−	4	bc	T
14	−	−	80% +	−	30% +	−	1	pc	NK
15	70% +	−	−	−	−	−	5	oc	T
16	−	−	80% +	−	40% +	−	15	pc	NK
17	−	−	80% +	−	70% +	−	11	mc+pc bg	NK

Abbreviations: −, negative; +, positive; MC, mitotic count (number of mitotic figures in 2.37 mm^2^); *TRG*, *T-cell receptor gamma* gene; pc, polyclonal; mc+pc bg, monoclonal plus polyclonal background; bc, biclonal; oc, oligoclonal; NK, NK cell; T, T-cell; NKT, NKT cell.

Clonality testing performed in all 17 patients showed clonality for *TRG* in 6 of 17 cases. The *IGH-VDJ* gene of all samples showed a polyclonal/negative/pseudoclonal result. A polyclonal/pseudoclonal *TRG* result was seen in the remaining 11 of 17 cases. Of the *TRG* clonal PCR results, 2 of 6 cases were monoclonal with a polyclonal background, and 2 cases each were biclonal (2 of 6 cases) and oligoclonal (2 of 6 cases). The 2 monoclonal cases with polyclonal background were represented by 1 CD3^+^ CD56^+^ granzyme B^+^ case, which was interpreted as NKT origin. The other case was CD3^−^ CD56^+^ granzyme B^+^. The 2 biclonal cases were CD3^+^ CD56^−^ cells—one of which was granzyme B^+^ and the other a granzyme B^−^ T-cell lymphoma. The oligoclonal samples were represented by CD3^+^ CD56^+^ granzyme B^−^ NKT cells and CD3^+^ CD56^−^ granzyme B^−^ T-cells, respectively. Detailed results of lymphocyte clonality PCR testing for *TRG* and proposed entity are shown in Table 2.

## Discussion

Despite the initial suspicion that FESF is always non-neoplastic, the current case series provides evidence that lymphoma can accompany eosinophilic inflammation and sclerosing fibrosis within the feline gastrointestinal tract. To our knowledge, thus far, there is only 1 case report of FESF and concurrent lymphoma. The lymphoma in that case emerged 7 months after surgical resection of an FESF-associated jejunal mass lesion and was concurrently in the jejunum and gastric serosa with typical FESF lesions. However, the lymphoma subtype of that case was not further specified.^35^

The etiology of FESF is currently unknown, and the potential role of pathogens observed within the lesions remains unclear. A genetic predisposition for the proliferative response stimulated by unknown antigens has been suggested.^4^ The inconsistent presence of infectious agents indicates that the pathogenesis of this proliferative, inflammatory disease may be multifactorial with potential contributing factors including food allergy or intolerance, and intestinal microbiota dysbiosis.^25^ Pathogens identified in previous studies include various bacteria,^4,17^ *Toxoplasma gondii*,^17^ phycomycetes,^9^ zygomycete fungi,^35^ and feline immunodeficiency virus.^4^ Other viral agents such as feline leukemia virus,^4^ feline coronavirus, and feline herpesvirus type 1 have not been detected thus far.^17^

Specific viruses have a carcinogenic potential in human and various animal species, and some (eg, retroviruses such as feline leukemia virus and feline immunodeficiency virus) may induce lymphoproliferative disease.^28^ An unidentified viral infection can be considered as a potential trigger of lymphoma following lymphoproliferation in this subset of FESF cases and additional investigative studies should be performed.

A review of complete blood count findings revealed that 12 of 15 cats in this case series had peripheral eosinophilia, which is also frequently demonstrated in cats with FESF.^4,5,17,24,30^

Intralesional eosinophils can be found regularly but variably in FESF, suggesting that they may play a central role in pathogenesis due to proinflammatory and immunomodulatory activities. Some authors suggest that FESF could also represent an unusual manifestation of the feline eosinophilic granuloma complex.^24^ Eosinophils can promote neoplastic growth by releasing various factors that stimulate angiogenesis, inflammation, and tissue remodeling. Furthermore, eosinophils can produce interleukin-5, which stimulates proliferation of eosinophils and other inflammatory cells.^32^ The presence of intralesional eosinophils in T-cell lymphoma, at least in the canine peripheral T-cell lymphoma,^33^ is described and there are few reports of T-cell lymphoma associated with severe hypereosinophilic syndrome in cats,^2,30^ demonstrating a close interaction between T-cells and eosinophils.

In general, mast cell neoplasm, which is commonly associated with eosinophils, must be considered as a differential diagnosis for intestinal round cell neoplasia. A case series documented intestinal sclerosing mast cell neoplasm in cats, which were accompanied by eosinophilic infiltrates,^12^ but some authors suggest that these cases may instead represent an inflammatory process, such as FESF.^7,27^

Blastic NK cell lymphoma/leukemia has been described in a cat,^13^ characterized by multisystemic infiltration of large, atypical round cells; however, eosinophil infiltration and fibrosis were not prevalent features. The neoplasm was in the subcutis, spleen, lymph nodes, bone marrow, and liver.

The NK cells are characterized by the expression of CD56 antigen and lack of CD3 labeling.^34^ CD57 antigen represents another NK cell–associated surface protein in humans.^29^ Cytotoxic markers like granzyme B protein can be found in NK cells as well as T-cells.^29^ The NKT cells are defined by expression of both CD56 and CD3 antigen.^34^ Summarizing the results of immunolabeling in our cases, the neoplastic lymphoid cells were interpreted as either NK cells (7 of 17 cases), NKT cells (7 of 17 cases), or T-cells (3 of 17 cases). The NK cells play a significant role in preventing intracellular invasion of microorganisms by directly killing them without prior sensitization. The intestinal immune system comprises innate lymphoid cells including NK cells.^14^ Some authors propose that NK cell proliferation restricted to the gastrointestinal tract is most probably induced by local inflammation or an immune reaction.^18^ The NK cells are involved in promotion and inhibition of fibrogenesis.^19^ In addition, eosinophils seem to be linked to immune-mediated fibrosis.^19^

In human medicine, intestinal NK cell lymphoproliferative disorders are uncommon and designated as NK cell enteropathy or extranodal NKT cell lymphoma.^14^ The NK cell enteropathy is a benign, localized, lymphoproliferative disorder of NK cells in the gastrointestinal tract with spontaneous regression or persistence.^14^ In contrast, extranodal NKT cell lymphoma is a heterogeneous, clinically aggressive neoplasm that is almost always associated with Epstein-Barr virus.^18,36^ Epstein-Barr virus has been serologically detected in cats, but the significance of infection is unclear.^20^

Of the 17 samples, 6 cases showed a clonal result for *TRG*. All *IGH* primer sets revealed no clonal result, so no cross-lineage phenomenon was present. The clonal *TRG* result was seen in the 3 T-cell lymphomas diagnosed (cases 12, 13, and 15), which showed a CD3^+^ CD56^−^ immunolabeling pattern. Of the 7 CD3^+^ CD56^+^ NKT cases, 2 cases (cases 3 and 6) showed a clonal result for *TRG*. A clonal result for all 7 NKT cases would be expected as T-cells are present, but to date, it is unclear whether feline lymphoma of NKT cells should demonstrate a clonal result for *TRG*. Six of 7 NK cell neoplasia cases showed no clonal result for *TRG* as expected. However, 1 CD3^−^ CD56^+^ granzyme B^+^NK case (case 17) had a monoclonal *TRG* result with a polyclonal background. This result should be evaluated further in an additional study.

If treated appropriately with a multimodal approach, long survival times have been reported for some cats with FESF.^17^ Remission has been described following corticosteroid administration.^1^ However, definitive therapeutic guidelines have not yet been established. In our study, most cats (9 of 13 cases for which outcome information was available) were euthanized within 3 months of the initial clinical diagnosis due to gastrointestinal disease, indicating an aggressive process with a relatively poor prognosis. Four cats treated with chemotherapy showed a survival time between 4 and 12 months, which suggests that this therapeutic method may be helpful in some cases. However, its efficiency would need to be reproduced and validated in larger studies.

This unique subtype of NK, NKT, or T-cell lymphoma should be considered as a differential diagnosis for gastrointestinal mass lesions and round cell tumors associated with FESF. We propose the term “eosinophilic sclerosing lymphoma” to intimate the similarities between FESF and this unique subtype of lymphoma. Further studies are required to better characterize the etiology, classification, outcome, and treatment response of this subset of FESF in order to optimize patient care.

## Supplemental Material

sj-pdf-1-vet-10.1177_03009858241281911 – Supplemental material for Feline eosinophilic sclerosing fibroplasia associated with T-/natural killer-cell lymphoma Supplemental material, sj-pdf-1-vet-10.1177_03009858241281911 for Feline eosinophilic sclerosing fibroplasia associated with T-/natural killer-cell lymphoma by Andrea Klang, Christof A. Bertram, Taryn A. Donovan, Linden E. Craig, Ingrid Walter, Birgitt Wolfesberger, Brigitte Degasperi, Elisabeth Baszler, Barbara C. Rütgen, Sabine E. Hammer and Andrea Fuchs-Baumgartinger in Veterinary Pathology
