# Supplementary material for: Feline eosinophilic sclerosing fibroplasia associated with T-/natural killer-cell lymphoma
Source: Vet Pathol. 2024 Sep 25;62(2):187–94. doi: 10.1177/03009858241281911 (PMC11874600; doi:10.1177/03009858241281911)
Supplement: sj-pdf-1-vet-10.1177_03009858241281911 – Supplemental material for Feline eosinophilic sclerosing fibroplasia associated with T-/natural killer-cell lymphoma [file sj-pdf-1-vet-10.1177_03009858241281911.pdf]

Supplemental Materials

Feline Eosinophilic Sclerosing Fibroplasia Associated with T-/Natural Killer- Cell Lymphoma

Andrea Klang, Christof A. Bertram, Taryn A. Donovan, Linden E. Craig, Ingrid Walter, Birgitt Wolfesberger, Brigitte Degasperi, Elisabeth Baszler, Barbara C. Rütgen, Sabine E. Hammer, and Andrea Fuchs-Baumgartinger

Supplemental Table S1. Antibody and pretreatment specifications.

Primary antibody						Pretreatment	Secondary antibody
Target protein	Clone	Host	Type	Provider	Dilution		
CD3	-	Rabbit	Polyclonal	Dako, Glostrup, DK	1:1000	HIER, pH6, Citrate	BrightVision, goat anti-rabbit IgG HRP, ready to use, ImmunoLogic, Duiven, NLD
CD20	-	Rabbit	Polyclonal	Abcam, Cambridge, UK	1:1000	HIER, pH8, Tris-EDTA	BrightVision, goat anti-rabbit IgG HRP, ready to use, ImmunoLogic, Duiven, NLD
CD56	1G4	Mouse	Monoclonal	LSBio, Lynnwood, WA	1:150	HIER, pH6, Citrate	BrightVision, goat anti-mouse IgG HRP, ready to use, ImmunoLogic, Duiven, NLD
CD57	VC1.1	Mouse	Monoclonal	Sigma Aldrich, St. Louis, MO	1:500	HIER, pH6, Citrate	BrightVision, goat anti-mouse IgG HRP, ready to use, ImmunoLogic, Duiven, NLD
Granzyme B	-	Rabbit	Polyclonal	Abcam, Cambridge, UK	1:400	HIER, pH6, Citrate	BrightVision, goat anti-rabbit IgG HRP, ready to use, ImmunoLogic, Duiven, NLD
MUM1	MRQ-8	Mouse	Monoclonal	Cell Marque, Rocklin, CA	-	HIER, pH8, Tris-EDTA	BrightVision, goat anti-mouse IgG HRP, ready to use, ImmunoLogic, Duiven, NLD

Abbreviation: HIER, heat induced epitope retrieval.
